# Quantification of bovine β-casein allergen in baked foodstuffs based on ultra-performance liquid chromatography with tandem mass spectrometry

**DOI:** 10.1080/19440049.2014.990994

**Published:** 2014-12-15

**Authors:** Qi Chen, Jingshun Zhang, Xing Ke, Shiyun Lai, Baohua Tao, Jinchuan Yang, Weimin Mo, Yiping Ren

**Affiliations:** ^a^ Zhejiang Provincial Center for Disease Control and Prevention, Hangzhou, China; ^b^ Zhejiang University of Technology, Hangzhou, China; ^c^ Waters Corporation, Milford, MA, USA

**Keywords:** allergens, bovine β-casein, tryptic digestion, isotope-labelled peptide, suspension digestion, baked foodstuffs

## Abstract

The quantification of allergens in food including baked food matrices is of great interest. The aim of the present study was to describe a non-immunologic method to quantify bovine β-casein using ultra-performance liquid chromatography tandem triple quadrupole mass spectrometry (UPLC-TQ-MS/MS) in multiple reaction monitoring (MRM) mode. Eight of 10 theoretical peptides from β-casein after tryptic digestion were compared and MRM methods were developed to determine five signature peptides. The peptide VLPVPQK was selected as the signature peptide for bovine β-casein because of the high sensitivity. A stable isotope-labelled internal standard was designed to adjust the instability of sample pre-treatment and ionisation caused by matrix effect. Using the present suspension digestion method, the native and denatured β-casein could be digested to release the signature peptide at the maximum extent. The UPLC-TQ-MS/MS method developed based on a tryptic signature peptide led to a reliable determination of bovine β-casein allergen in baked food matrices at a low quantitation level down to 500 μg kg^–1^ with a satisfactory accuracy (< 8.9%) and recovery (98.8% ± 2.6% to 106.7% ± 3.0%).

## Introduction

About 2% of the general population suffers from food allergies (Ring et al. 2001). Allergy to cow’s milk is considered as one of the most common food allergies (Bock & Atkins 1990). β-Casein is one of the major allergens in cow’s milk proteins (IUIS Allergen Nomenclature 2014). Food allergic consumers should completely avoid eating food containing allergenic ingredients, because even a minimal amount of allergenic ingredient can trigger adverse reactions, including life-threatening allergic reactions (Ring et al. 2001; Brockow & Ring 2009). However, there were some cases in which allergenic ingredients were unrecognised or not declared on the product labels (hidden allergens) (Anibarro et al. 2007; Van Hengel 2007), which put food-allergic consumers at great risk. In order to prevent these accidents from happening, food manufacturers and food testing laboratories are responsible for ensuring that products are free of allergens or labelled accordingly.

The detection methods of food allergens include PCR and ELISA as the traditional techniques and MS-based methods as emerging techniques (Poms et al. 2004b; Monaci et al. 2006; Johnson et al. 2010).

PCR is based on the detection of DNA with a specific base pair sequence to an allergenic food (Hird et al. 2003; Scaravelli et al. 2008). PCR methods are highly specific to the allergen of interest. However, PCR methods are sensitive to the presence of food matrix components and the food processing history. Furthermore, PCR methods are not able to distinguish between by-product proteins (such as milk and egg) from tissue proteins (such as beef and chicken). The PCR method is seldom used for egg or milk allergen testing in processed foods.

The ELISA is based on the interaction of an allergenic protein with its antibody and is fast, easy to use and sensitive. The LOD ranges from 1 to 2 mg kg^–1^ (Holzhauser & Vieths 1999; Weber et al. 2007). ELISA methods have been validated at an international level and are widely adopted as standard methods in many countries (Poms et al. 2005). However, the antibodies used in ELISA methods are generally expensive. ELISA methods are also susceptible to false-positive results because of the cross-reactivity with interfering matrix components, and false-negative results due to protein modifications, such as aggregation and the Maillard reaction of proteins in food processing (Paschke & Besler 2002; Downs & Taylor 2010).

In recent years, LC-MS-based methods have emerged as new approaches for protein and allergen analysis. Czerwenka et al. (2007) and Ren et al. (2010) developed LC-MS methods for the qualitative and quantitative analysis of native proteins. However, these methods were not suitable for the baked foods because proteins might be denatured during processing. Heick et al. (2011) developed a MRM-based LC-MS method for qualitative screening of the characteristic peptides of allergens after tryptic digestion. Although the method had high sensitivity and selectivity during the screening of allergen proteins, it could not be applied for the quantitative determination of allergens in baked foodstuffs. Using the same approach, Lutter et al. (2011) developed a quantitation method for four unheated cow’s milk allergens in food products. They used stable isotope-labelled peptides that were homologues for peptides derived from bovine milk allergens as the internal standards (ISs). The IS was spiked into samples after the tryptic digestion to alleviate the matrix effect in MS detection. However, it could not correct the loss of analytes in sample preparation. Moreover, they just analysed the native but not the denatured allergen proteins. Zhang et al. (2012) employed a peptide analogue of a signature peptide derived from α-lactalbumin with a slight modification on the amino acid sequence as the IS. The IS was added into the sample prior to the tryptic digestion, so that it could normalise any variation in the tryptic digestion process. However, this IS could cause non-identical behaviours in MS detection because it had a similar but not an identical sequence with the target analyte.

The aim of this paper is (1) to establish a sensitive and selective method for the determination of bovine β-casein based on its tryptic signature peptides; (2) to design stable isotope-labelled internal standards; and (3) to optimise the sample preparation method in order to digest the soluble and insoluble β-casein after baking.

## Materials and methods

### Chemicals and reagents

Ammonium bicarbonate (NH_4_HCO_3_), dithiotheritol (DTT), iodoacetamide (IAA), hydrogen chloride (HCl) and bovine β-casein (purity > 98%) were obtained from Sigma-Aldrich (St. Louis, MO, USA). Acetonitrile (ACN) and formic acid were purchased from Merck (Darmstadt, Germany). All reagents used were of analytical or HPLC grade. Proteomics grade trypsin was from Yaxin Biotechnology Co., Ltd (Shanghai, China). Stable isotope-labelled [^13^C_5_, ^15^N]-valine (V*) and [^13^C_6_, ^15^ N]-leucine (L*) were purchased from Sigma-Aldrich. The stable isotope-labelled peptides VL*PV*PQK (IS1) and QSVLSLSQSKVL*PV*PQKAVPYPQRD (IS2) were synthesised by ChinaPeptides Co., Ltd (Shanghai, China). Trypsin was dissolved and diluted in 1 mM HCl, while other chemicals were prepared using 50 mM NH_4_HCO_3_ with no further purification. Water generated from a Milli-Q Gradient A 10 system (Millipore, Bedford, MA, USA) was used in all experiments. Human milk was obtained from the local hospital and used as IS3.

The study was approved by the Ethics Committee of the College of Biosystem Engineering & Food Science, Zhejiang University.

### Apparatus

#### UPLC-TOF and data analysis

LC was carried out using the ACQUITY UPLC System equipped with ACQUITY UPLC binary solvent manager, ACQUITY UPLC sample manager and ACQUITY UPLC column manager (Waters, Milford, MA, USA). Separation of peptides was performed on an ACQUITY UPLC BEH300 C18 column (1.7 μm particle size, 2.1 × 100 mm) (Waters), equipped with a guard column of the same material. Mobile phase A was water with 0.1% formic acid and mobile phase B was acetonitrile with 0.1% formic acid. The mobile phase flow rate was 0.3 ml min^–1^. The injection volume was 5 μl. The column temperature was 40°C. The LC elution program was a linear gradient from 3% B to 40% B in 10 min, ramped up to 100% B in 0.1 min, then held at 100% B for 2 min, returned back to 3% B in 0.1 min and equilibrated at 3% B for 2.8 min. The total injection cycling time was 15 min.

Time of flight mass spectrometry (TOF-MS) detection was performed on a Synapt G2 HDMS equipped with an electrospray ion (ESI) source (Waters). All data were acquired in the electrospray positive ion (ESI+) mode with MS^E^ mode. Details of TOF conditions were as follows: capillary voltage, 3 kV; sampling cone voltage, 25 V; extraction cone voltage, 4 V; source temperature, 100°C; desolvation temperature, 400°C; cone gas flow, 30 l h^–1^; desolvation gas flow, 800 l h^–1^; ramp trap collision energy, 15–35 V; and lockspray reference compound, leucine-enkephalin.

The acquired data were analysed using ProteinLynx Global Server version 2.5 software with the followed settings: mode, electrospray-MS^E^; lockmass for charge 1, 556.2771 Da; minimal fragment ion matches per peptide, 2; minimal fragment ion matches per peptide, 5; allowed missed cleavage, 1; fixed modifications, carbamdomethyl C; and variable modifications, oxidation M. The databank was imported from UniProt Knowledgebase (http://www.uniprot.org).

#### UPLC-TQ-MS/MS

The same UPLC system, column and solvents were used for quantification with UPLC-TQ-MS/MS. The LC elution program was the same as given in the ‘UPLC-TOF and data analysis conditions’ section.

Quantitative detections were performed on a Xevo-TQ-MS equipped with an ESI source (Waters). The conditions of TQ-MS/MS were set as follows: capillary voltage, 3.5 kV; source temperature, 120°C; desolvation temperature, 400°C; cone gas flow, 30 l h^–1^; desolvation gas flow, 800 l h^–1^; and argon collision gas pressure, 3 × 10^–3^ mbar. The precursor ion, product ion and their optimal MRM parameters are shown in Table 2.

### Preparation of standard solutions

The stock standard solutions of β-casein (1 mg ml^–1^) and IS2 solution (1 μg ml^–1^) were prepared in 50 mM NH_4_HCO_3_. All solutions can be stored at −20°C for 3 months. Working standard solutions that ranged from 1 to 10 mg kg^–^^1^ and from 10 to 100 mg kg^–1^ were prepared from these stock solutions and diluted step by step with 50 mM NH_4_HCO_3_ for the quantification of the samples with low β-casein concentration (< 100 mg kg^–1^) or high β-casein concentration (> 100 mg kg^–1^), respectively. An aliquot (100 μl) of each working standard solution was mixed with 10 μl IS2 solution and 900 μl 50 mM NH_4_HCO_3_. These working standard solutions were mixed with the same amount of DTT and IAA solutions as in the ‘Tryptic digestion and peptide extraction’ section and the same tryptic digestion procedure was carried out.

### Sample preparation

#### Preparation of laboratory-made β-casein-positive/negative samples

The β-casein-positive food samples were prepared as follows: 125 g wheat flour, 2.5 g salt, 25 g peanut oil, 1 g sodium bicarbonate and 50 ml 315.72 mg l^–1^ β-casein solution were mixed in a kneading bowl. The mixture was kneaded for 1 h by a food processor to ensure the even distribution of the β-casein in the dough. The dough was rolled out to a thin flat dough sheet (approximately 4 mm thickness) and cut into small pieces (4 × 4 cm square). They were baked for 25 min at 170°C in a static oven. The dough was weighed before and after baking to calculate the moisture loss. Therefore, the final concentrations of β-casein in the baked food samples could be calculated. All ingredients were purchased from a local supermarket.

The β-casein negative food samples were prepared in the same process as the β-casein positive samples, except that 50 ml of water were used in place of 50 ml of β-casein solution.

#### Tryptic digestion and peptide extraction

A total of 0.2 g of sample with high β-casein concentration (> 100 mg kg^–1^) or 2 g sample with low β-casein concentration (< 100 mg kg^–1^) were weighed in a 50 ml tube. The sample was mixed with 20 ml 50 mM NH_4_HCO_3_ and homogenised with a homogeniser. A total of 1 ml of the suspension was transferred into a vial and spiked with 10 μl IS2 solution (1 μg  ml^–1^ in 50 mM NH_4_HCO_3_) and 10 μl DTT solution (50 mM in 50 mM NH_4_HCO_3_). The mixture was incubated in a 60°C water bath for 30 min. After the sample was cooled to RT, 10 μl IAA solution (150 mM in 50 mM NH_4_HCO_3_) was added to react for 30 min at RT in the dark. The solution was then added to trypsin (200 μg ml^–1^ in 50 mM NH_4_HCO_3_) and incubated for 1 h at 37°C. The tryptic digestion was stopped by adding 5 μl of formic acid. The insoluble substances in tryptic hydrolysate were removed by centrifuging at 13000 *g* for 10 min. The supernatant was filtered by 0.22 μm nylon filter before analysis.

## Results and discussion

### Search and selection of signature peptides

Trypsin was chosen as the protease because the cleavage occurred at the carboxyl side of the amino acids lysine or arginine with very high specificity. An *in silico* tryptic digestion of bovine β-casein was performed with the PeptideMass tool provided by UniProt. Ten of the 16 theoretical digestion products could be used as the potential candidates for a signature peptide (Table 1). The other six theoretical digestion products, including two tripeptides, two dipeptides and two single amino acids, were not reported due to lack of amino acid sequence specificity.

**Table 1.** Theoretical peptides of simulated tryptic digestion and detectable peptides using UPLC-TOF.

Number	Peptide	Position	Matched product ions	Peptides score^a^
1	YPVEPFTESQSLTLTDVENLHLPLPLLQ SWMHQPHQPLPPTVMFPPQSVLSLSQSK	114–169	16	7.74
2	IHPFAQTQSLVYPFPGPIPNSLPQNIPPLT QTPVVVPPFLQPEVMGVSK	49–97	8	7.40
3	ELEELNVPGEIVESLSSSEESITR	2–25	Undetectable	
4	DMPIQAFLLYQEPVLGPVR	184–202	31	8.58
5	FQSEEQQQTEDELQDK	33–48	Undetectable	
6	AVPYPQR	177–183	2	6.91
7	VLPVPQK	170–176	7	7.79
8	EMPFPK	108–113	5	7.47
9	GPFPIIV	203–209	10	8.07
10	EAMAPK	100–105	5	7.03

Note: ^a^Peptide scores were calculated by ProteinLynx Global Server Version 2.5 software.

A 100 μg ml^–1^ β-casein solution was digested by trypsin and injected into the UPLC-TOF.

After comparing the acquired data and the protein sequence from UniProt using ProteinLynx Global Server software, eight of the 10 theoretical peptides were detected (Table 1). The coverage of the searched peptides from the total bovine β-casein sequence is 70.0%.

**Figure 1.**
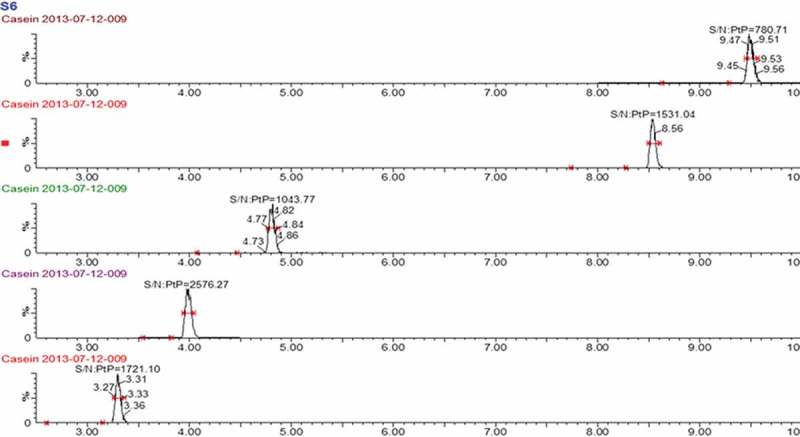
MRM chromatograms of the quantitative product ion spectra from the selected precursors of the target peptides with the signal-to-noise ratio, from top to bottom: DMPIQAFLLYQEPVLGPVR, GPFPIIV, EMPFPK, VLPVPQK and AVPYPQR.

Based on the parameters obtained from the UPLC-TOF analysis, including the electronic charge, molecular weight and retention time of each peptide, the product ions of peptide candidates were searched using the UPLC-TQ-MS/MS with the daughter scan mode. Peptides 1 and 2 were not selected as they both showed poor chromatographic resolution because of long sequences (Table 1). Peptide 10 was not selected because the signal of its product ions was extremely low during UPLC-TQ-MS/MS analysis. After optimisation of the MS parameters (see the ‘UPLC-TQ-MS/MS conditions’ section), three product ions for each of the other five peptides (peptides 4 and 6–9 in Table 1) with the best sensitivity were selected for the establishment of MRM methods (Table 2). The specificity and selectivity of the five signature peptides were evaluated *in silico* and *in vitro*. The *in silico* evaluation was performed using the BLAST tool. However, BLAST can be carried out only with the peptides contain more than eight amino acid in their sequence such as peptide 4 in Table 1. The BLAST with peptides 6, 7 and 9 was performed after adding arginine or lysine before the N-terminal of the sequences. Otherwise, the signature peptides could not be produced after tryptic digestion. Peptide 8 could not be blasted due to the short sequences. Peptides 4, 6, 7 and 9 could be found only in β-casein from various mammalian species. The BLAST result showed that the four peptides would not exist in other proteins in the Uniprot. However, the current proteomic databases could not be considered exhaustive in terms of food ingredients. Therefore, an *in vitro* experiment was carried out with the often used ingredients in baked foods, such as wheat flour, soybean, coconut, cacao and chicken egg. The often-used ingredients in baked foods were pre-treated and analysed as described in the ‘Tryptic digestion and peptide extraction’ and ‘UPLC-TQ-MS/MS conditions’ sections. No chromatographic peaks of the peptides were identified and detected. However, the chromatographic peaks of these peptides appeared when a β-casein solution was also digested and analysed using the present method. The peptide VLPVPQK was finally selected as the signature peptide for β-casein in view of the signal intensity and signal-to-noise ratio (Figure 1). Other four peptides candidates were also qualitative analysed to enhance the confidence of the present of β-casein.

**Table 2.** MRM parameters of the precursor ion, product ion, cone voltage, collision energy and type of fragment for each candidate signature peptide and ISs.

Peptide	Precursor ion (*m/z*)	Cone voltage (V)	Product ion (*m/z*)	Collision energy (eV)	Fragment
EMPFPK	374.9	15	146.1	25	y1
			243.2	15	y2
			487.3	10	y4
VLPVPQK	391.0	15	213.5	12	b2
			371.8	18	y3
			568.3	12	y5
AVPYPQR	416.9	15	175.1	26	y1
			330.7	10	y5
			400.2	18	y3
DMPIQAFLLYQEPVLGPVR	730.8	20	428.0	29	y4
			737.0	29	y7
			867.0	26	y8
GPFPIIV	742.9	40	441.3	27	y4
			512.3	27	b5
			625.4	23	b6
VL*PV*PQK (IS1)	397.5	15	220.3	12	b2
			371.8	18	y3
			574.3	12	y5
VMPVLK (IS3)	344.0	15	231.2	10	b2
			260.2	17	y2
			456.3	12	y4

### Comparison of IS strategies

There have been a few approaches to find the most suitable internal standard strategy. Arsene et al. (2008) and Pritchard et al. (2011, 2013) demonstrated IS strategies with stable isotope-labelled protein. It was considered as a potential golden standard of the IS strategy because it was always added into the sample at the first step of sample preparation and theoretically could normalise all variation during the whole experimental process. However, the high cost of stable isotope-labelled protein has limited the scope of application. Lutter et al. (2011) designed a stable isotope-labelled IS homologue of the signature peptide, which was added after the tryptic digestion. This strategy could normalise the variation of the ionisation during the MS detection. However, it could not correct for the loss of analytes during the sample pre-treatment processes. Zhang et al. (2012) used an improved IS strategy. They designed a peptide containing a similar amino acid sequence to the signature peptide of bovine α-lactalbumin in the middle with the extension of several amino acids from the C- and N-end homologue to the original sequence of bovine α-lactalbumin. This IS was added prior to sample extraction and the tryptic digestion to correct any loss of analytes during sample pre-treatment. The tryptic digestion process was generally a robust and reliable process. However, if there were any interfering compounds, such as ovomucoid from eggs, in the food matrix that made the tryptic digestion incomplete, the quantification would be inaccurate. Since this IS relied on tryptic digestion to be converted, any change in the digestion reaction would affect the IS in the same way as the target analyte. All variations in the tryptic digestion reaction could be normalised by this IS. If any trypsin inhibitor existed in the sample, there would be no IS be converted. Therefore, such IS not only could normalise the variation during the tryptic digestion reaction but also could be used as an indicator for the presence of trypsin inhibitor.

The present study investigated three IS strategies. The first strategy (IS1) was a stable isotope-labelled peptide, VL*PV*PQK. It had the same amino acid sequence of bovine β-casein signature peptide, VLPVPQK. The second strategy (IS2) was a stable isotope-labelled long peptide, QSVLSLSQSKVL*PV*PQKAVPYPQRD. It had the amino acid sequence of bovine β-casein signature peptide in the middle with several additional amino acids on both ends of the native sequence of bovine β-casein. This IS can theoretically, similar to the stable isotope-labelled protein, adjust the variation by tryptic digestion and ionisation, but the cost was much cheaper. The third strategy (IS3) was human β-casein. Although IS1 and IS2 were homologues of bovine β-casein sequence, they were much smaller molecules than the bovine β-casein protein. They might have different physical and chemical properties from the bovine β-casein protein. Human β-casein might act as a good candidate for IS in the quantification of bovine β-casein because they had a similar molecular size and physicochemical properties. The peptide VMPVLK was chosen as the signature peptide of human β-casein. There was no interference detected between signature peptides of bovine and human β-casein. The shortcoming of human β-casein (IS3) was that its signature peptide VMPVLK had a different sequence from the signature peptide VLPVPQK of bovine β-casein, which could cause non-identical behaviours in MS detection (Table 1).

**Figure 2.**
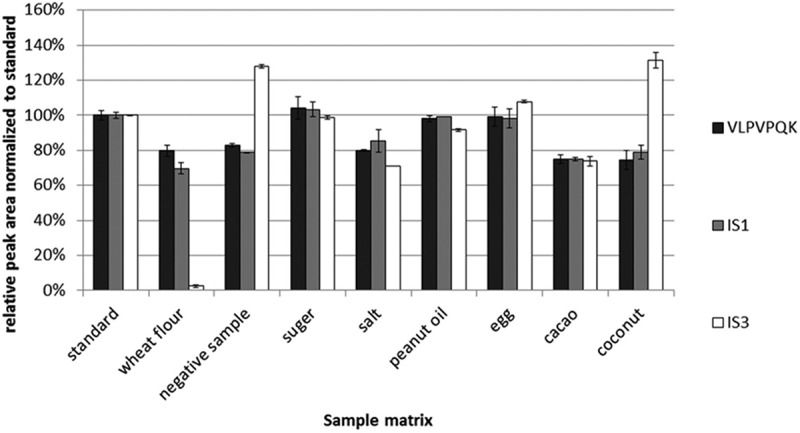
Comparison of the relative peak areas normalised to a standard of IS1, IS3 and a signature peptide in different food matrices (*n* = 3). A total of 10 µg ml^–1^ bovine β-casein solution and 1:100 diluted human milk were digested respectively and combined with 0.25 µg ml^–1^ IS1 solution at the ratio of 1:1:1 (v/v/v) to form a digest mixture. The negative sample and commonly used baking ingredients (wheat flour, sugar, salt, peanut oil, hen’s eggs, cacao and coconut) were treated identical with human and bovine β-casein with trypsin, and mixed in a ratio of 1:1 (v/v) with the digest mixture. The standard solution was prepared by mixing the digest mixture and water in the ratio of 1:1 (v/v), and its peak area was set as 100% abundance.

Two experiments were designed to investigate the performance of these IS strategies. In the first, the bovine β-casein and the human milk (IS3) were digested separately without the presence of any food matrix. Then they were combined and mixed with IS1 to form a digestion mixture. The negative sample and common baking ingredients, such as wheat flour, sugar, salt, peanut oil, hen’s eggs, cacao and coconut, were also digested separately in the same way as the proteins. Aliquots of the digest mixture were spiked into each food matrix and analysed to evaluate the effects of the food matrix on the MS detection. The standards were prepared by mixing the digest mixture with water. The peak areas of the quantitative signature peptide and ISs in food matrix samples were compared with those in the standards and normalised to the standards. The resulting relative peak areas were used to compare the ionisation efficiency of the quantitative signature peptide and ISs in the presence of food matrices (Figure 2). IS2 was not used in this study because its digestion product was identical to IS1.

IS1 and signature peptide (VLPVPQK) showed similar behaviour in the presence of negative sample and baking ingredients (Figure 2). The IS3 (human β-casein) behaved differently in the MS detection when compared with the signature peptide. The IS3 peak area was significantly enhanced in the presence of the negative sample and the coconut, while it was significantly reduced in the presence of wheat flour. It is not surprising that IS3 is not suitable to be used as the IS since its signature peptide is different from the bovine β-casein’s signature peptide.

In the second experiment, bovine β-casein was mixed with each food matrix first, then each sample mixture was spiked with an IS (IS1 or IS2) separately. Each of these food matrix samples was digested according to the procedure described in the ‘Tryptic digestion and peptide extraction’ section. Bovine β-casein was also spiked with each of the ISs at the same concentrations as the food matrix samples, without the presence of any food matrix, and labelled as the standards. They were digested and analysed in the same way as the food matrix samples. The peak areas of the signature peptide (VLPVPQK) and the ISs in the food matrix samples were compared with those corresponding peak areas in the standard mixtures. The relative peak areas of the signature peptide and ISs in food matrix samples were employed to evaluate the effect of food matrix on the overall analysis process, including the tryptic digestion, extraction and MS detection.

**Figure 3.**
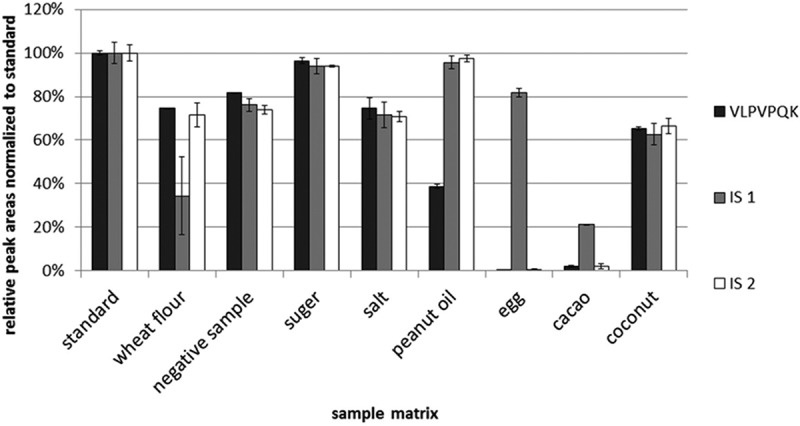
Relative peak areas normalised to a signature peptide standard and IS1, IS2 in different food matrices (*n* = 3). A total of 0.1 g negative sample and commonly used baking ingredients (wheat flour, sugar, salt, peanut oil, hen’s eggs, cacao and coconut) were weighed into the Eppendorf caps and mixed with 100 µl of 10 µg ml^–1^ bovine β-casein. After spiking of 10 µl IS1 (0.25 µg ml^–1^) and IS2 (1 µg ml^–1^) into the caps, the mixtures were digested as described in the ‘Tryptic digestion and peptide extraction’ section. The solutions of bovine β-casein and ISs at the same concentration were digested as a standard, and its peak area was set as 100% abundance.

The results (Figure 3) showed that the relative peak areas of signature peptide (VLPVPQK) in the presence of different food matrix were similar to the results of the first experiment (Figure 2) except in egg, cacao and peanut oil. There was almost no signature peptide detected in the presence of egg because egg contained ovomucoid, which was known for its trypsin inhibitor property. Cacao is rich in polyphenols, which can interact with proteins to form complex in solution. It was speculated that polyphenols in cacao might inhibit the digestion process because of their interaction with the bovine β-casein and the trypsin. Figure 3 also showed that in the presence of peanut oil, the content of signature peptide was about 40% of the signature peptide in the standard mix. This could not be simply explained by inhibition of the tryptic digestion because the IS2 was not affected by the presence of peanut oil in the same tryptic digestion. Only signature peptide was affected by the peanut oil, possibly because the non-identical behaviour between protein and IS peptide in an oily sample. The digestion steps releasing the corresponding signature peptides from target proteins require the cleavage sites to be solvent accessible for proteolysis to occur. Nevertheless, the high oil contents in food matrices affect the solvent accessible levels of target proteins and IS in different extent. The usage of stable isotope-labelled protein might solve this problem (Pritchard et al. 2014). However, it is worth noting that the negative sample in this study contained about 16% peanut oil. The relative peak area of signature peptide in negative sample in experiment 2 was almost identical to the results of experiment 1. All these results showed that the signature peptide of β-casein (VLPVPQK) could work well with the negative sample and the wheat flour, sugar, salt and coconut. This signature peptide might not work well with baked foodstuff containing egg, cacao or high content of oil.

**Figure 4.**
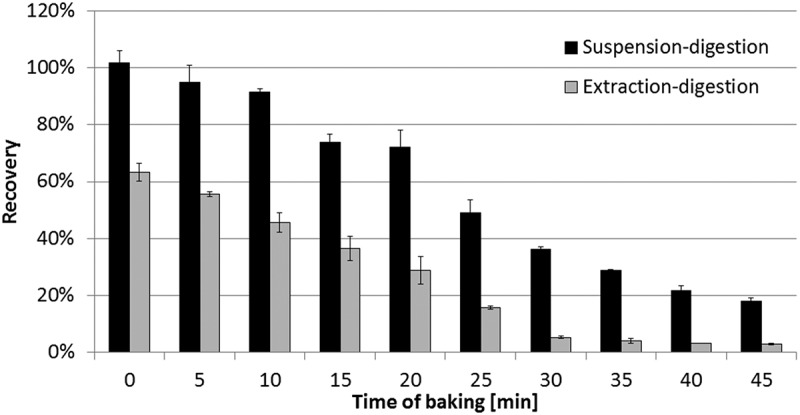
Recoveries of extraction and suspension digestion: black, suspension digestion; and white, extraction digestion (*n* = 3).

The major difference between IS1 and IS2 is that IS2 requires tryptic digestion to be converted to IS1 (VL*PV*PQK) while IS1 does not require digestion. The relative peak areas of IS1, IS2 and the signature peptide of β-casein in different food matrices were evaluated. IS1 peak area changed in a similar way to the signature peptide in four food matrices (negative sample, sugar, salt, coconut), while IS2 peak areas changed in a similarly way to the signature peptide in seven food matrices (except the peanut oil) (Figure 3). It seemed that IS2 was a better candidate for IS than IS1. It was also interesting that in the presence of egg and in cacao, a little amount of IS2 was detected. This indicated that IS2 could be used as a marker to monitor the tryptic digestion reaction. If there was a little amount of IS2 detected, there might be a problem in the tryptic digestion, and this methodology might not work for that sample.

The results of these two experiments showed that the signature peptide worked well in baked foodstuff containing wheat flour, sugar, salt and coconut. For baked foodstuff containing egg, cacao or a high level of oil, this signature peptide might not work for the quantification of bovine β-casein. IS2 was the best IS strategy for this method.

### Optimisation of the sample pre-treatment method

There were few published papers about allergenic protein extraction and detection in baked foodstuff. The major problem was the extraction efficiency of allergenic protein from baked foodstuff because a part of the proteins were denatured to an insoluble form after heating. The common allergen analysis methods, including the commercial ELISA kits, were based on the extraction of the target proteins from the sample matrix before quantification (extraction digestion). The protein that was not extracted could not be analysed by ELISA (Poms et al. 2004a; Downs & Taylor 2010) or digested to be quantified by UPLC-MS/MS because the heat process led to a loss in solubility.

In this work, four MS-friendly buffers were investigated from the literature and tested with the positive sample (see the ‘Sample preparation’ section). In addition, the sample extracted by water was quantified as a reference sample. The buffer from the ELISA kits or from the literature that described the ELISA method were not chosen because they contain a harmful detergent for the MS. The IS2 was added into the samples before extraction, and the extract was digested analogous to the ‘Tryptic digestion and peptide extraction’ section and quantified. The recovery was calculated using a relative method with IS2 as IS.

As described in Table 3, methods 1, 2 and 4 did not exhibit a significantly better recovery compared with water extraction. Although extraction method 3 provided a higher recovery, the peak areas and absolute recovery of signature peptides and IS were only approximately 10–20% of the values from the extract with water. This indicated that the neutral or alkaline buffer was better than the acidic buffer for extracting bovine β-casein. In particular, the buffers could provide an extraction efficiency similar to water, which might only extract the soluble bovine β-casein but not the insoluble β-casein. European Directive 2000/13/EC (European Commission 2000) clearly states that the presence of an allergenic ingredient should be indicated on the label, even if the ingredient was modified during processing. These extraction methods could not satisfy the requirement for the quantification of bovine β-casein in baked food matrices because the level of allergenic proteins might be underestimated.

**Table 3.** Comparison of recovery obtained with the different extraction method.

	Buffer	Extraction method	Recovery^a^	Reference
1	50 mM NH_4_HCO_3_, 1 M urea, pH 8.0	2 g sample added with 20 ml preheated (60°C) buffer, homogenised, shake for 15 min	24.4% ± 0.8%	Lutter et al. (2011)
2	Tris-buffer, pH 8.2	2 g sample added with 20 ml buffer, homogenised, incubated at 60°C for 3 h	21.5% ± 0.5%	Heick et al. (2011)
3	1 M guanidine hydrochloride, 20 mM DTT, 10 mM sodium citrate, pH 6.75	1 g sample added with 25 ml buffer, homogenised, shake for 30 min	35.9% ± 0.8%	Monaci et al. (2010)
4	4.3 mM Na_2_HPO_4_, 1.4 mM KH_2_PO_4_, 137 mM NaCl, 2.7 mM KCl, pH 7.4	2 g sample added with 20 ml buffer, homogenised, incubated at 4°C for 3 h^b^	19.9% ± 0.8%	Kim et al. (2002)
5	Water	2 g sample added with 20 ml water, homogenised, shake for 3 h	23.1% ± 1.4%	
6	Method described in the ‘Tryptic digestion and peptide extraction’ section		50.8% ± 1.6%	

Notes: ^a^Recovery = detected β-casein/spiked β-casein*100%.

^b^The ratio of sample to water was increased from 1:3 in the literature to 1:10.

The complete extraction of bovine β-casein from baked food matrix was the key issue for quantification. We propose here a novel one-step process (suspension digestion). That is to say, the resulting suspension was directly digested after samples were mixed with extraction solution. The soluble β-casein, as well as the insoluble β-casein, could be extracted and quantified. A part of the β-casein was insoluble after heat processing because of the polymerisation of β-casein and/or covalent binding between β-casein and matrix. However, there was less chance that the modifications occurred in the sequence of VLPVPQK than in whole β-casein sequence, because VLPVPQK was composed of seven amino acids accounting for 3.4% of the total bovine β-casein sequence. Most VLPVPQK could be extracted from insoluble β-casein after tryptic digestion. The recovery of bovine β-casein in a positive sample could be improved to 50.8% ± 1.6%. Although the recovery of the method was not perfect, the result illustrates the fact that the suspension-digestion process was significantly better than extraction-digestion process.

In order to verify the advantage of suspension digestion, dough was prepared according to the ‘Sample preparation’ section. The dough squares were baked at 170°C for 0–45 min. The theoretical concentration of bovine β-casein in the baked product was calculated after excluding the loss of water. Every three squares of each bake time were homogenised together and digested using the extraction-digestion process (50 mM NH_4_HCO_3_ as buffer, 1 g of sample extracted with 10 ml of buffer, centrifuge, 1 ml of supernatant digested analogous to the ‘Tryptic digestion and peptide extraction’ section) or suspension-digestion process (homologous to the ‘Tryptic digestion and peptide extraction’ section), respectively.

The first dough squares (baking time = 0 min) were analysed directly after kneading without baking. However, approximately 36.7% of bovine β-casein could not be extracted and quantified using the extraction-digestion process, probably because they were physically adsorbed onto the matrix during kneading. This result indicated that the extraction-digestion process could not extract all β-casein in dough. However, the recovery of β-casein of the first dough squares was 101.9% ± 4.2% using the suspension-digestion process (Figure 4). As speculated, the insoluble β-casein could be maximally digested by suspension digestion.

The recoveries using extraction digestion reduced along with increasing the baking time. It might be caused by the increase of the denatured bovine β-casein after baking. The detectable β-casein by extraction digestion was possibly still in a native and soluble form which survived from heating. The recoveries using suspension digestion also reduced along with increasing the baking time, but were always higher than extraction digestion. The different of recoveries between extraction and suspension digestion could be considered as the insoluble β-casein. This part of the β-casein was denatured only slightly. The modification of the molecule occurred in other sequence than the signature peptide VLPVPQK or in the 3D structure of the proteins. Maybe that is why more signature peptide was produced after digestion in suspension though the protein was not extracted. Before the depletion of soluble bovine β-casein (baking time from 0 to 25 min), the content of insoluble β-casein was maintained at 33–45%. However, there was still a undetectable part of β-casein by suspension digestion. The percentage of undetectable β-casein was increased during baking. It was hard to identify the existence form of this part of β-casein. Probably some modification of the β-casein might occur after baking just on the sequence of the signature peptide VLPVPQK, although the chance was theoretically very little. It might also indicate that the signature peptide VLPVPQK was absent as the β-casein protein had been completely degraded.

### Method validation

The method validation, including the linearity, LOQs, recovery and precision, was carried out with a standard solution and a laboratory-made negative sample (see the ‘Sample preparation’ section).

The linear equations for the concentration sequences that ranged from 1 to 10 and from 10 to 100 µg ml^–1^ were *y* = 0.0904*x* – 0.001 and *y* = 0.0969*x* – 0.139, respectively. Good linearity and coefficients of determination (*r* ^2^ > 0.99) were obtained over both concentration ranges.

The LOQ of the present method was evaluated by spiking the standard in the laboratory-made negative sample with the concentration of 1 mg kg^–1^. It was calculated as the lowest concentration that provided a signal-to-noise ratio of 10. The LOQ was 500 µg kg^–1^.

As for the recovery test, three levels of β-casein (4, 20 and 100 mg kg^–1^) were spiked in the negative sample. After pre-treatment analogously with the ‘Tryptic digestion and peptide extraction’ section, the samples were quantified and their concentrations calculated using the theoretical concentrations. The recoveries were 98.8% ± 2.6%, 106.7% ± 3.0% and 99.7% ± 2.7%, respectively.

The precision study was carried out with a laboratory-made positive sample at 20 mg kg^–1^. This experiment was repeated 11 times within a day for the intra-day precision test, and performed with three replicates each day, continuously for 5 days, for the inter-day test. The intra- and inter-day precisions were 3.9% and 8.9%, respectively.

## Conclusions

A UPLC-TQ-MS/MS method was established for the quantification of a trace amount of bovine β-casein allergen. The MRM methods of five candidates of signature peptides were developed, and the peptide VLPVPQK was finally selected due to the highest sensitivity. After comparing three IS strategies, a stable isotope-labelled long peptide, QSVLSLSQSKVL*PV*PQKAVPYPQRD (IS2), was finally chosen because it could adjust the instability of not only the ionisation, but also sample pre-treatment. Furthermore, the extraction efficiency was enhanced using the suspension-digestion process which provided better recoveries compared with the classical protein extraction method. All the validation results showed that the developed method was accurate, sensitive and selective.

## Appendix: Abbreviations

V*: stable isotope-labelled [^13^C_5_, ^15^N]-valine.
L*: stable isotope-labelled [^13^C_6_, ^15^N]-leucine.
IS: internal standard; IS1 = VL*PV*PQK.
IS2: QSVLSLSQSKVL*PV*PQKAVPYPQRD.
IS3: human milk.
